# Commentary: Defining long-term survivors in metastatic lung cancer: insights from a Delphi study in Spain

**DOI:** 10.3389/fonc.2025.1633958

**Published:** 2025-07-18

**Authors:** Javier-David Benitez-Fuentes, Pablo Oliva Marin, Beatriz Grau Mirete, Samer Alhallak Alhallak, Paula Rodríguez Payá, Asia Ferrández Arias

**Affiliations:** ^1^ Medical Oncology Department, Hospital General Universitario de Elche, Elche, Spain; ^2^ Primary Care Department, Centro de Salud Integrado de Santa Pola, Departamento de Salud de Elche-Hospital General, Santa Pola, Spain; ^3^ Primary Care Department, Centro de Salud de Torrellano, Departamento de Salud de Elche-Hospital General, Torrellano, Spain

**Keywords:** metastatic lung cancer, survivorship, primary care, health system, immunotherapy, multidisciplinary care

## Introduction

Carcereny et al. present a valuable Delphi consensus that aims to standardize what it means to be a “long-term survivor” (LTS) in metastatic lung cancer (mLC). Such a definition is especially relevant now that immunotherapy and targeted treatments have reshaped the prognosis in advanced disease (1). Their threshold of ≥3 years of overall survival (or ≥2 years of progression-free survival) offers a reference point for both clinical practice and future research (1). This nationwide expert effort reflects the growing body of literature demonstrating that metastatic lung cancer is no longer invariably a short-term illness (2).

The authors appropriately highlight the oncological challenges of detecting second neoplasms, immune-mediated toxicities and the broader psychosocial and rehabilitative needs these long-term survivors face (1). Many individuals living with advanced cancer experience fatigue, fear of recurrence, financial toxicity, and difficulties reintegrating into work or community life (3). Based on experiences in other tumor settings, such as breast or colorectal cancer, where extended survival has been observed for decades, collaboration between oncologists and primary care physicians (PCPs) can strengthen survivorship care (4).

Carcereny et al. report that 70.7% of their Delphi panel agreed the follow-up of long-term survivors of lung cancer should not be carried out in primary care, while there was no clear consensus about follow-up being conducted exclusively in specialized units (agreement 53.7%) (1). In this commentary, we differ from their position regarding primary care follow-up. We suggest that their conclusion likely reflects a cautious assessment of the current limitations in primary care infrastructure. However, we advocate transitioning toward a primary care-led or shared-care follow-up model after an initial oncology-led period. Greater investment and improved resources could enable primary care providers to better address the needs of mLC survivors, ultimately enhancing the resilience of the health system.

## Why the follow-up of long-term survivors of lung cancer should be carried out in primary care or in shared-care models

There is evidence that primary care–led or co-led models may improve long-term outcomes, quality of life, and possibly cost-efficiency. PCPs and other primary care providers such as nurses and social workers hold deep knowledge of their patients’ comorbidities, social contexts, and lifestyles, allowing them to approach survivorship holistically. This continuity of care could be critical in ensuring that screening for secondary malignancies, and management of chronic conditions are integrated into the patient’s care plan (5–8).

Patients with a history of advanced malignancies may develop a variety of post-treatment effects, some of which are subtle, cumulative, and often mistaken for normal aging or other comorbidities. PCPs, thanks to frequent and less specialized visits, are well positioned to detect new or evolving symptoms—ranging from respiratory complaints to changes in mental health—offering pathways back into oncology if alarm signs arise (8). Regular interactions with primary care may also foster a preventive approach as PCPs and their allied primary care teams are accustomed to recommending lifestyle changes and screening for other diseases, reinforcing beneficial behaviors that reduce the risk of recurrences and secondary tumors (8, 9).

When managed carefully, primary care–led or shared-care follow-up can be cost-effective, reducing avoidable specialist appointments and shortening diagnostic delays (10). Ongoing primary care involvement in cancer survivor follow-up could enhance satisfaction and timeliness of care, partly because PCPs and primary care teams address a broad range of issues in a single visit (8). Evidence from integrated care models demonstrates that transitioning cancer survivors from specialty to primary care maintains cancer-specific outcomes while simultaneously enhancing management of general health issues, providing better supportive care, and reducing overall healthcare costs compared to specialist-led models (8).

Importantly, strengthening primary care may also improve the performance, efficiency, and resilience of the entire health system (6, 11). A comparative analysis underscored how countries with strong primary care foundations consistently outperform others in terms of better overall health outcomes, lower costs, and greater equity in healthcare provision (12). Similarly, a comprehensive assessment argued that health systems built on a robust primary care infrastructure exhibit increased resilience, responding more effectively to population needs both during routine care and health crises such as the COVID-19 pandemic (13).

## Discussion

Implementing primary care–led follow-up requires addressing several concerns. Studies have noted potential drawbacks, for example, the possibility of delayed recognition of cancer recurrence if surveillance is less intensive, limited oncology-specific expertise among some PCPs, and many survivors’ preferences to continue seeing oncology specialists for follow-up (14–18). Indeed, certain high-risk survivors, such as those with very aggressive tumors or complex treatment-related complications, may need ongoing specialist oversight, given the specialized knowledge required to manage their surveillance and late effects (17). Such concerns were perhaps reflected in the Delphi panel led by Carcereny et al., which concluded that follow-up of long-term survivors should not occur in primary care, possibly due to perceived limitations in current primary care capabilities.

Nevertheless, we disagree with this conclusion, emphasizing instead that primary care’s role could—and should—be expanded through increased investment and resources. The optimal approach may be an initial oncology-led follow-up tailored according to treatment complexity and emerging evidence, transitioning to primary care or shared-care follow-up for routine survivorship visits if the patient remains stable. Such a model requires clear guidelines, support and training for PCPs and their multidisciplinary primary care teams and streamlined pathways for prompt re-access to oncology services when and if necessary. Making primary care a fully capable partner requires an investment in infrastructure and additional workforce training. Adequate resources, such as enhanced electronic health records, improved training in recognizing late immunotherapy side effects, and robust communication channels with oncology, will ensure that responsibilities are not shifted without support.

A summary of the key benefits supporting primary care-led follow-up is provided in **Table 1**.

**Table 1:** Advantages of primary care–led follow-up for long-term survivors of metastatic lung cancer, key reasons and their justifications.

Reason	Justification
Holistic Approach	Comorbidities, lifestyle, and psychosocial issues addressed in tandem
Continuity of Care	Long-standing, trust-based relationships with patients enable personalized support
Local Accessibility	Reduced travel burdens and improved adherence through community-based follow-up
Cost-Efficiency	More efficient resource use; frees oncology services for complex/relapsed cases
Early Symptom Detection	Timely detection of second tumors or late toxicities; rapid referral to oncology when needed
Preventive Services	Routine provision of smoking cessation, vaccinations, nutrition counseling, and mental health care
Health System Resilience	Investment in primary care infrastructure benefits all patients, not just cancer survivors

The Delphi study by Carcereny et al. makes a valuable contribution by defining “long-term survivor” in metastatic lung cancer and underscoring the complexities of caring for these survivors. Their consensus strongly validates the importance of specialized monitoring for toxicity and disease progression (1). Yet an equally vital role for primary care teams, including PCPs, nurses, social workers, and other primary care professionals is now in sharper focus. By integrating the expertise of oncologists during the initial phases following treatment—tailored to clinical complexity and evolving evidence—with the holistic, patient-centered care provided subsequently by primary care, health services can comprehensively address patients’ comorbidities, psychosocial needs, and preventive health strategies, provided patient stability is maintained. Although such a model requires infrastructure, workforce expansion, and ongoing collaboration, the long-term dividends, improved cost efficiency, better patient access, enhanced early detection of complications, and a stronger health system overall, are likely substantial.
